# Pattern of prolactin secretion after administration of gonadotropin-releasing hormone agonist at the preovulatory phase of intrauterine insemination cycles

**DOI:** 10.1590/S1516-31802005000600010

**Published:** 2005-11-01

**Authors:** Mario Cavagna, João Carlos Mantese, Gilberto da Costa Freitas, Artur Dzik, Jonathas Borges Soares, Yaron Hameiry, Vicente Mario Izzo, José Aristodemo Pinotti

**Keywords:** Gonadorelin, Ovulation induction, Infertility, Insemination artificial, Prolactin, Gonadorrelina, Indução da ovulação, Infertilidade, Inseminação artificial, Prolactina

## Abstract

**CONTEXT AND OBJECTIVE:**

Administration of a gonadotropin-releasing hormone (GnRH) agonist at the preovulatory phase is an option for triggering ovulation in assisted reproductive technology cycles. The aim of this work was to investigate the pattern of prolactin secretion after the administration of a single dose of GnRH-agonist at the preovulatory phase.

**DESIGN AND SETTING:**

Descriptive study at a tertiary referral center.

**PARTICIPANTS:**

Fifteen normally ovulating patients undergoing ovarian stimulation for intrauterine insemination were studied.

**METHODS:**

Ovarian stimulation was carried out using human menopausal gonadotropin (intra-muscular 75 IU daily). When at least one follicle reached 17 mm (observed echographically), 0.5 mg of buserelin acetate was administered. Blood samples were taken to determine prolactin concentrations, at the time of agonist injection and 4, 8, 12, 24 and 48 hours later.

**RESULTS:**

A statistically significant increase in serum levels of prolactin was observed 4, 8 and 12 hours after GnRH-agonist administration, with a peak at 8 hours.

**CONCLUSION:**

The administration of a single dose of GnRH-agonist at the preovulatory phase in patients undergoing ovarian stimulation performed with human menopausal gonadotropin causes a significant increase in serum prolactin levels.

## RESUMO

**CONTEXTO E OBJETIVO:**

A administração de um agonista do hormônio liberador das gonadotropinas no momento pré-ovulatório é uma opção para desencadear a ovulação nos ciclos de reprodução assistida. O objetivo deste trabalho é investigar o padrão da secreção de prolactina após a administração de dose única de agonista no momento pré-ovulatório.

**TIPO DE ESTUDO E LOCAL:**

Estudo descritivo realizado em um centro de referência terciário.

**PARTICIPANTES:**

15 pacientes normo-ovulatórias submetidas a indução da ovulação para inseminação intra-uterina.

**MÉTODOS:**

A estimulação ovariana foi realizada com gonadotropinas da mulher menopausada (75 UI por via intramuscular diariamente). Na presença de pelo menos um folículo de 17 mm (observado por ultra-sonografia), administrou-se 0,5 mg de acetato de buserelina. Foram colhidas amostras de sangue para determinação dos níveis de prolactina no momento da injeção do agonista e 4, 8, 12, 24 e 48 horas depois.

**RESULTADOS:**

Observou-se aumento significante nas concentrações séricas de prolactina 4, 8 e 12 horas após a administração do análogo, com um pico após 8 horas.

**CONCLUSÃO:**

A administração de uma dose única de análogo agonista no momento pré-ovulatório de pacientes estimuladas com gonadotropinas da mulher menopausada promove aumento significante nas concentrações séricas de prolactina.

## INTRODUCTION

In protocols for ovarian stimulation for the treatment of infertility, gonadotropin-releasing hormone (GnRH) agonist analog has been used as an alternative to human chorionic gonadotropin for triggering ovulation.^1^ Administration of the analog at the preovulatory phase induces an endogenous surge of luteinizing hormone (LH) and follicle-stimulating hormone (FSH), and the effect may be more physiological than that of exogenous human chorionic gonadotropin.^2^ This study was designed to investigate whether the pattern of prolactin secretion is also affected by the administration of GnRH-agonist.

## METHODS

After approval by the ethical committee of the Women's Health Reference Center, São Paulo, fifteen patients between 23 and 40 years of age (mean: 31.5 years) who were undergoing intrauterine insemination gave their informed consent and participated in the study. All the patients had ovulatory menstrual cycles, as judged from FSH and LH determinations on day 3 of cycle, timed endometrial biopsies and midluteal phase serum progesterone determinations. The indications for intrauterine insemination were cervical hostility and mild male factor.

Basal FSH concentrations ranged from 3.9 to 10.8 mIU/ml and basal LH levels ranged from 2.4 to 9.6 mIU/ml. Prolactin concentrations were measured between days 21 and 23 of the previous menstrual cycle, and ranged from 6.2 to 17.8 ng/ml.

Ovarian stimulation was performed with human menopausal gonadotropin (hMG) at a dose of 75 IU intramuscularly, daily, commencing on day 3 of the menstrual cycle. When at least one follicle was 17 mm or more at its maximum diameter, the patients received a single dose of buserelin acetate, 0.5 mg subcutaneously. Serum samples for the determination of prolactin were collected at the time of agonist injection and 4, 8, 12, 24 and 48 hours later. Estradiol levels were measured in the first sample. Prolactin levels were analyzed in duplicate, using the fluorimetric enzyme immunoassay system (Stratus, Baxter Diagnostics Inc.).

The significance of differences between prolactin concentrations was determined by using variance analysis (ANOVA). Probability values < 0.05 were considered to be significant.

## RESULTS

All the fifteen women progressed to the point of buserelin administration after stimulation with hMG. The number of follicles ³ 17 mm at their greatest diameter ranged from 1 to 4 and, at the time of GnRH-agonist administration, serum estradiol levels ranged from 205.9 to 608.0 pg/m (Table 1). Serum concentrations of prolactin (means ± standard deviation, SD) presented a statistically significant increase (p < 0.0001) 4, 8 and 12 hours after GnRHagonist administration, with a peak after 8 hours and returning to the initial levels after 24 hours. Statistically, prolactin levels at the times zero, +24 and +48 were no different from each other. Likewise, the levels at the times +4 and +12 were no different from each other. All of these levels were different from the concentrations obtained at the time +8 (Table 2).

**Table 1.** Outcome from ovarian stimulation in 15 women, using human menopausal gonadotropin (intramuscular 75 IU)

Patient	Age	Follicles ≥ 17 mm	Estradiol*
1	23	3	415.00
2	25	4	608.00
3	27	2	251.60
4	28	2	305.55
5	30	3	422.35
6	30	1	205.90
7	31	2	327.20
8	31	2	293.00
9	32	3	446.40
10	33	2	240.70
11	35	1	239.15
12	36	2	393.50
13	36	3	428.75
14	36	2	305.00
15	40	2	292.80

* *Mean estradiol values (pg/ml) at the time of agonist administration.*

**Table 2.** Prolactin levels (mean ± SD) after gonadotropin-releasing hormone (GnRH) agonist administration (n = 15)

Time (hours)	Prolactin (ng/ml) Mean ± SD	Minimum value (ng/ml)	Maximum value (ng/ml)
0	14.98 ± 6.05	5.95	29.65
4	21.87 ± 11.45	7.40	52.50
8	32.07 ± 13.68	16.30	63.70
12	26.81 ± 12.91	12.75	64.10
24	16.27 ± 7.32	4.00	30.70
48	13.86 ± 6.72	4.10	32.15

*Variance analysis (ANOVA): F = 13.89; p < 0.0001 (0 = 24 = 48) ≠ 8 ≠ (4 = 12); SD = standard deviation.*

## DISCUSSION

The significant increase in prolactin levels observed after the administration of the GnRH-agonist may be due to direct inhibition of hypothalamic dopaminergic activity. There is evidence^3^ that the administration of GnRH causes a decrease in plasmatic dopamine, epinephrine and norepinephrine levels. It is postulated that the reduction in plasma catecholamine concentrations represents direct action by GnRH in pre-synaptic receptors of central neurons, since this effect can be cancelled by prior treatment with bromocriptine.^3^ Zhang and Yen^4^did not find detectable changes in plasma levels of catecholamines after administration of GnRH. It is possible, however, that the measurement of plasma catecholamines may not be sensitive towards reflecting subtle changes in central dopaminergic activity. The GnRH-agonist induced prolactin secretion may be a consequence of paracrine factors capable of stimulating prolactin release. However, the possibility of direct action on the lactotrophs cannot be ruled out. As seen with other hypothalamic hormones, GnRH and its analogs seem to have several effects, and this investigation has shown that one of them is prolactin release. There is, to our knowledge, no published report of a rise in prolactin concentrations determined by human chorionic gonadotropin administration. It seems that the increase in prolactin serum concentrations does not cause deleterious effects on the outcome from infertility treatment.

## CONCLUSION

The administration of GnRH-agonist at the preovulatory phase to trigger the ovulation process causes a significant rise in serum prolactin concentrations. This has never been observed with the administration of human chorionic gonadotropin for the same purpose.
